# Resilience and Cognitive Function in Patients With Schizophrenia and Bipolar Disorder, and Healthy Controls

**DOI:** 10.3389/fpsyt.2018.00279

**Published:** 2018-06-29

**Authors:** Mengjie Deng, Yunzhi Pan, Li Zhou, Xudong Chen, Chang Liu, Xiaojun Huang, Haojuan Tao, Weidan Pu, Guowei Wu, Xinran Hu, Zhong He, Zhimin Xue, Zhening Liu, Robert Rosenheck

**Affiliations:** ^1^Hunan Key Laboratory of Psychiatry and Mental Health, Department of Psychiatry, Second Xiangya Hospital of Central South University, China National Clinical Research Center on Mental Health Disorders, China National Technology Institute on Mental Disorders, Hunan Technology Institute of Psychiatry, Mental Health Institute of Central South University, Changsha, China; ^2^Department of Psychiatry, First Affiliated Hospital of Jinan University, Guangzhou, China; ^3^Department of Psychiatry, Hunan Brain Hospital, Changsha, China; ^4^Medical Psychological Center, Second Xiangya Hospital, Central South University, Changsha, China; ^5^Department of Radiology, Second Xiangya Hospital of Central South University, Changsha, China; ^6^Department of Psychiatry, Yale University, New Haven, CT, United States

**Keywords:** schizophrenia, bipolar disorder, resilience, cognitive function, CD-RISC

## Abstract

**Background:** This study compared adaptive resilience among patients with schizophrenia, bipolar disorder, and healthy controls, and examined the relationship of resilience to cognitive function.

**Methods:** A sample of 81 patients diagnosed with schizophrenia, 34 with bipolar disorder, and 52 healthy controls completed the Connor-Davidson Resilience Scale (CD-RISC) and cognitive tests of verbal comprehension, executive functioning, and working memory. Paired comparison of diagnostic groups on CD-RISC and cognitive tests was conducted. Linear regression was used to identify the independent association of clinical diagnoses and neurocognition with resilience deficits.

**Results:** Both patient groups showed significantly lower CD-RISC scores and poorer cognitive function than healthy controls and the schizophrenia group scored lower than bipolar group on these measures as well. CD-RISC scores were positively correlated with all three cognitive measures in the entire sample but not within the diagnostic subgroups. Multiple regression analysis showed differences in CD-RISC between diagnostic groups were not mediated by differences in these three measures of neurocognition.

**Discussion:** Schizophrenia and bipolar disorder are associated with impairments in both resilience and cognitive function but the impairment in resilience appears to be independent of deficits in cognitive function measured here and may reflect unmeasured dimensions of cognitive function, other impairments or environmental factors.

## Introduction

It has increasingly been recognized that poor adaptation to social context (1, 2) and more specifically deficient resilience, the dynamic process of adapting to and functioning in the face of adversity, is impaired in patients with both schizophrenia and bipolar disorder (3). Mizuno et al. in particular, found that Resilience Scale total scores were significantly lower in patients with schizophrenia and bipolar disorder than in healthy controls, although the difference between patient groups was not significant (4). A research demonstrated that patients who recently received a diagnosis of schizophrenia spectrum disorder showed less resilience than healthy controls (5). And patients with chronic non-remitted schizophrenia also showed lower resilience scores than healthy controls (6). Choi et al. found that resilience level was significantly lower in euthymic patients with bipolar disorder than in healthy controls, and the number of past depressive episodes significantly correlated negatively with resilience (2). Additionally, individuals at clinical high risk for psychosis also have shown impairment in resilience compared to healthy individuals (7). Altogether, both schizophrenia and bipolar disorder patients show deficits in resilience, especially patients who recently received diagnosis of schizophrenia spectrum disorder. Even in the patients with remitted state of bipolar disorder, resilience level were significantly lower than healthy individuals.

Cognitive deficits are considered to be hallmarks of both schizophrenia and bipolar disorder. Lee et al., for example, found that both schizophrenia and bipolar patients exhibited impairment in non-social cognitive function as compared to healthy controls, with the lowest levels of non-social cognitive function in schizophrenia (8). While previous studies have suggested that higher levels of cognitive performance may be associated with greater resilience in healthy adults (9), the direct relationship between resilience and cognitive function in either schizophrenia or bipolar disorder has not been studied.

More recently, it has been hypothesized that cognitive function may influence the course of serious mental illness through its impact on resilience which, in turn, may mediate functional outcomes (10). The aim of the present study was to compare resilience and cognitive function in patients diagnosed with schizophrenia, patients diagnosed with bipolar disorder, and a sample of healthy controls and to determine if observed deficits in resilience are attributable to deficits in cognitive function. Based on the research published to date, we hypothesize that (1) resilience and cognitive function correlate with each other in patients with schizophrenia and bipolar disorder; and (2) that the deficits in resilience among these patients are attributable to the impairment in cognitive function.

## Methods

### Subjects

Using the Structured Clinical Interview-Patient version for DSM-IV, 81 patients who met diagnostic criteria for schizophrenia; and 34 with bipolar disorder were recruited from the inpatient and outpatient units of the Department of Psychiatry of the Second Xiangya Hospital of Central South University, Changsha, China. Inclusion criteria included: (1) age between 16 and 34 years old; (2) met DSM-IV criteria for schizophrenia or bipolar I disorder; (3) at least 9 years of education for both groups; and (4) Han Chinese ethnicity. Exclusion criteria included (1) a lifetime history of loss of consciousness for more than 1 h due to head trauma; and (2) history of receiving electroconvulsive therapy; (3) history of alcohol or substance abuse except nicotine; (4) chronic neurological disorders or debilitating physical illness.

In addition to the two groups with confirmed diagnoses, 52 healthy controls, whose first-degree relatives had no history of psychiatric disorders, no past or current psychiatric disorder according to DSM-IV Axis I disorder criteria, were recruited. The inclusion and exclusion criteria for healthy controls were the same as those for patients except that they did not meet the DSM-IV criteria for any psychiatric disorders.

All participants signed informed consent after all procedures were fully explained, according to procedures approved by the ethics committee of the Second Xiangya Hospital of Central South University.

### Clinical information

Clinical characteristics of patients with schizophrenia were assessed using the Scale for Assessment of Positive Symptoms (SAPS) (11) and the Scale for Assessment of Negative Symptoms (SANS) (12), while patients with bipolar disorder were assessed using the Hamilton Depression Rating Scale (HAMD) (13) and Young Mania Rating Scale (YMRS) (14).

### Connor-Davidson Resilience Scale

The Connor-Davidson Resilience Scale (CD-RISC, Chinese version) is a 25-item self-rating scale measuring the degree of individual resilience, and it assesses resilience to both social and non-social sources of adversity (15). CD-RISC has been tested in the general population, as well as in clinical samples, and demonstrated sound psychometric properties, with good internal consistency and test-retest reliability (15). The Chinese language version of CD-RISC is as reliable and valid as the original English version as a measure of the resilience construct in Chinese society, and the alpha coefficient value for the Chinese version of CD-RISC was 0.91 (3). In completing this measure, the subject is directed to respond to each item with reference to the previous month. Scoring of the full 25-item scale is based on summing the scores from all items, all of which carry a 5-point range of responses, as follows: not true at all (0), rarely true (1), sometimes true (2), often true (3), and true nearly all of the time (4). The full range is therefore 0-100, with higher scores reflecting greater resilience (15).

### Cognitive assessments and tasks

Southwick et al. have reported that the ability to cognitively reframe adversity and the capacity to extract meaning from adverse situations might be associated with resilience (16). Moreover, Nonsocial cognition is thought to be a determinant of functioning in both schizophrenia and bipolar disorder (8, 17). In this study, the non-social cognitive functioning was assessed with the Information subscale of Wechsler Adult Intelligence Scale-Chinese Revised (WAIS-CR) as well as with tests of verbal fluency (VF) and the N-back task (N-back).

The Information subscale of WAIS-CR is an individually administered measure of intelligence, intended for adults aged 16–89, which is used to test verbal comprehension (18), we used it to test the cognitive function of verbal comprehension here. It includes 29 items addressing general information which are assumed to have opportunities to acquire within their culture, in this case the culture of contemporary China. No specialized of academic information is required. Each item is scored 0–1 point.

The test of verbal fluency, interpreted as a measure of “executive functioning,” requires participants to say the names of different animals as many as possible during a 1 min test interval. Each word marks 1 point (19). Higher scores on both of the cognitive assessments reflect better cognitive functioning. The test validity of verbal ability and executive control functions has been proven because the test not only demands the ability of verbal retrieval and recall, but also the self-monitoring and inhibition of responses in a certain period of time (20).

The letter N-back test was used to assess working memory (21). The task involves two conditions involving exposure to a series of letters: in the “0-back” condition, subjects are asked to press a button each time they see the letter x; while in the “2-back” condition, they are asked to press a button when the letter presented is identical to the letter they were shown two letters earlier in the sequence. Each letter is displayed for 500 ms with an inter-stimulus interval of 1500 ms. Each block comprised 20 stimuli containing 7 targets and is preceded by an instruction shown for 2 s. During the resting periods, a cross is presented in the center of the screen for 20 s. Stimulation blocks and resting periods alternate with a total of four 2-back and four 0-back blocks. The final score represents the accuracy rate with higher rates reflecting better working memory.

### Statistical analysis

Demographic characteristics across the three groups were compared with one-way analysis of variance (ANOVA) for continuous variables and Chi-square tests for categorical variables. The CD-RISC and cognitive function tests were similarly compared across the three groups with analysis of covariance (ANCOVA) with adjustment for any significant differences between groups on demographic variables. Paired comparisons of CD-RISC scores and cognitive function tasks across groups (i.e., the general information subscale of WAIS-CR, VF, and N-back) were conducted using ANCOVA, with adjustment for multiple comparisons (LSD methods were used for *post-hoc* paired comparisons) to identify significant differences between pairs of groups.

Pearson correlation was then conducted on the whole sample to identify the significance of the correlation between CD-RISC and measures of cognitive function and repeated to evaluate the association between CD-RISC and cognitive function measures within each of the three groups.

To determine whether measures of cognitive function mediated the association of diagnosis and CD-RISC, dummy coded variables were created to represent each diagnostic group leaving healthy controls as the reference group in a set of regression analysis (22). In the first step, multivariate linear regression was used to confirm the association of each of the two diagnostic groups and CD-RISC as compared to healthy controls net of any effects of sociodemographic characteristics found to be significantly different between groups. In the second step, linear regression was conducted to indentify the association between diagnostic groups and neurocognitive measures by controlling the sociodemographic characteristics found to be significantly different between groups. In the third step, linear regression model in the first step was then repeated with measures of cognitive function added as covariates. We hypothesized that with neurocognitive measures added to the multiple regression analysis the dummy coded diagnostic variables would be reduced to non-significance or would remain significant with a substantially smaller coefficient than in the models without neurocognitive measures, demonstrating that differences in neurocognitive function could account for differences between diagnostic groups in CD-RISC. A *p*-value of < 0.05 was considered statistically significant. Statistical analysis was performed using SPSS Statistics Version 19.

## Results

### Demographic data, resilience and cognitive function performance

There were no significant differences between the three groups on age or on between the patient groups on illness duration. However significant differences were observed on years of education (*F* = 25.84, *p* < 0.001), gender (χ^2^ = 6.46, *p* = 0.04), marital status (χ^2^ = 9.45, *p* = 0.02) and employment (χ^2^ = 63.46, *p* < 0.001) which were included as covariates in subsequent analyses (Table 1).

**Table 1:** Demographic data, clinical information and cognitive function in three groups.

**Characteristics**	**1. Schizophrenia (*n =* 81)**	**2. Bipolar disorder (*n =* 34)**	**3. Healthy controls (*n =* 52)**	**Statistical values**	**Comparison**
Age (years)^a^	22.79 ± 3.94	22.71 ± 2.90	22.08 ± 2.25	*F =* 0.79, *p* = 0.45	
Education years^a^	11.67 ± 2.56	12.91 ± 2.81	14.77 ± 1.88	*F =* 25.84, *p* < 0.001	
Gender, n (%)				*X^2^* = 6.46, *p* = 0.04	
Male	52(64.20%)	17(50.00%)	22(42.31%)		
Female	29(35.80%)	17(50.00%)	30(57.69%)		
Marital status, n (%)				*X^2^* = 9.45, *p* = 0.02	
Married	5(6.17%)	5(14.71%)	0		
Single/divorced/widowed	76(93.83%)	29(85.29%)	52(100%)		
Employment, n (%)				*X^2^* = 63.46, *p* < 0.001	
Employed	15(18.52%)	8(23.53%)	9(17.65%)		
Student	17(20.99%)	14(41.18%)	40(78.43%)		
Unemployed	49(60.49%)	12(34.29%)	2(3.92%)		
Illness duration (months)^a^	33.38 ± 35.94	38.13 ± 46.67	/	/	
SAPS^a^	22.37 ± 17.89	/	/	/	
SANS^a^	37.75 ± 29.47	/	/	/	
HAMD^a^	/	9.59 ± 8.03	/	/	
YMRS^a^	/	9.06 ± 10.43	/	/	
CD-RISC^a^	48.64 ± 17.22	61.44 ± 18.18	69.83 ± 11.70	*F =* 17.89, *p* < 0.001	1 < 2 < 3^b^
**COGNITIVE FUNCTION**					
WAIS-CR^a^	16.14 ± 5.53	19.07 ± 4.99	21.76 ± 4.99	*F =* 3.94, *p* = 0.02	1 < 2 < 3^c^
Verbal fluency^a^	14.38 ± 4.60	17.68 ± 4.55	20.83 ± 5.06	*F =* 12.87, *p* < 0.001	1 < 2 < 3^d^
N-back^a^	0.46 ± 0.23	0.51 ± 0.24	0.69 ± 0.21	*F =* 10.01, *p* < 0.001	1 < 3, 2 < 3^e^

a *Values shown as mean ± SD*.

b *1 vs. 2: p < 0.001, 1 vs. 3: p < 0.001, 2 vs. 3: p = 0.02*.

c *1 vs. 2: p = 0.007, 1 vs. 3: p < 0.001, 2 vs. 3: p = 0.02*.

d *1 vs. 2: p = 0.001, 1 vs. 3: p < 0.001, 2 vs. 3: p = 0.003*.

e *1 vs. 2: p>0.05, 1 vs. 3: p < 0.001, 2 vs. 3: p < 0.001*.

CD-RISC resilience scores were significantly different among three groups (*F* = 17.89, *p* < 0.001), and remained so after adjustment for covariates (*p* < 0.001). Paired comparisons, showed CD-RISC total scores to be significantly different between each pair of groups, with schizophrenia patients showing the lowest scores, followed by bipolar patients and healthy controls (Table 1).

Measures of cognitive performance also showed significant differences across groups on the information subscale of the WAIS-CR (*F* = 3.94, *p* = 0.02), on VF (*F* = 12.87, *p* < 0.001) and on the N-back test (*F* = 10.01, *p* < 0.001), even after controlling for the covariates (all *p* < 0.05). Paired comparisons again showed significant differences on measures of cognitive performance on all paired comparisons between all three groups on the information subscale of the WAIS-CR and on Verbal Fluency with the single exception of the schizophrenia and bipolar disorder groups on the N-back (Table 1).

### Correlation between resilience level and cognitive performance

We found significant and positive correlations between CD-RISC and all three measures of cognitive function in the whole sample (WAIS-CR: *r* = 0.36, *p* < 0.001; VF: *r* = 0.33, *p* < 0.001; N-back: *r* = 0.27, *p* < 0.001). But subgroup analyses showed no consistently significant relationships within the schizophrenia and bipolar groups. In contrast, significant differences were observed between neurocognitive measures and resilience in the healthy controls (WAIS-CR: *r* = 0.46, *p* < 0.01; VF: *r* = 0.39, *p* < 0.01; N-back: *r* = 0.30, *p* < 0.05) (Table 2).

**Table 2:** Correlation between resilience and cognitive function among three groups.

	**CD-RISC**			
	**Whole Samples**	**Schizophrenia**	**Bipolar disorder**	**Healthy controls**
WAIS-CR	0.36^***^	0.21	−0.14	0.46^**^
VF	0.33^***^	−0.007	−0.002	0.39^**^
N-back	0.27^***^	0.07	−0.10	0.30^*^

* *p < 0.05*;

** *p < 0.01*;

*** *p < 0.001*.

### Mediation analysis with multiple regression

In Tables 3-1–3-3, regression analysis revealed that, in the first step, the initial regression analysis showed both dummy coded diagnostic variables was negatively associated with resilience, even after controlling for sociodemographic measures that were different among groups (schizophrenia: β = −0.55, *t* = −5.84, *p* < 0.001; bipolar disorder: β = −0.18, *t* = −2.19, *p* = 0.03). In the second step, schizophrenia was negatively associated with the WAIS-CR (β = −0.21, *t* = −2.62, *p* = 0.01), VF (β = −0.46, *t* = −4.97, *p* < 0.001) and N-back (β = −0.42, *t* = −4.37, *p* < 0.001), and bipolar disorder was negatively associated with the N-back (β = −0.28, *t* = −3.26, *p* = 0.001) but no other measures of cognitive function. In the third and final step, after addition of all three neurocognitive measures to the regression analysis in the first step, both dummy coded diagnostic variables remained significant with only minimal reduction in the magnitude of their coefficients with one exception. In the mediation analysis of the N-back on the relationship between bipolar disorder and resilience, bipolar disorder showed no significant correlation with resilience. Measures of neurocognition were not significantly associated with resilience in the final step.

**Table 3-1:** The mediating effect of WAIS-CR on the relationship between diagnostic groups and resilience.

**Model**	***t***	***p***	***R^2^***
Y = −0.55X_1_	−5.84	<0.001	0.28
−0.18X_2_	−2.19	0.03	
M = −0.21X_1_	−2.62	0.01	0.45
−0.05X_2_	−0.65	>0.05	
Y = −0.51X_1_	−5.39	<0.001	0.26
−0.17X_2_	−2.11	0.04	
+0.16M	1.76	>0.05	

*X_1_, schizophrenia; X_2_, bipolar disorder; M, WAIS-CR; Y, CD-RISC*.

**Table 3-2:** The mediating effect of VF on the relationship between diagnostic groups and resilience.

**Model**	***t***	***p***	***R^2^***
Y = −0.55X_1_	−5.84	<0.001	0.28
−0.18X_2_	−2.19	0.03	
M = −0.46X_1_	−4.97	<0.001	0.31
−0.16X_2_	−1.95	>0.05	
Y = −0.52X_1_	−5.16	<0.001	0.25
−0.17X_2_	−2.05	0.04	
+0.06M	0.70	>0.05	

*X_1_, schizophrenia; X_2_, bipolar disorder; M, VF; Y, CD-RISC*.

**Table 3-3:** The mediating effect of N-back on the relationship between diagnostic groups and resilience.

**Model**	***t***	***p***	***R*^2^**
Y = −0.55X_1_	−5.84	<0.001	0.28
−0.18X_2_	−2.19	0.03	
M = −0.42X_1_	−4.37	<0.001	0.24
−0.28X_2_	−3.26	0.001	
Y = −0.53X_1_	−5.28	<0.001	0.25
−0.17X_2_	−1.94	>0.05	
+0.05M	0.69	>0.05	

*X_1_, schizophrenia; X_2_, bipolar disorder; M, N-back; Y, CD-RISC*.

## Discussion

This study compared adaptive resilience and cognitive performance between patients diagnosed with schizophrenia, bipolar disorder and a comparison group of healthy controls. Both patient groups showed lower level of resilience and impaired cognitive performance than controls on bivariate analyses, and schizophrenia patients exhibited more severe impairment compared than patients with bipolar disorder. Bipolar patients showed an intermediate level of resilience between those with schizophrenia and healthy controls on measures of both resilience and cognitive performance. While there was significant correlation between resilience and cognitive measures using data from the entire sample including the healthy controls group there were no significant relationships within diagnostic schizophrenia or bipolar subgroups. The mediation analysis, furthermore, showed no evidence that the cognitive measures assessed in this study mediated the relationship between clinical diagnoses and resilience.

The finding that the measure of resilience was significantly lower in patients with schizophrenia and bipolar disorder compared to healthy controls, is consistent, albeit more robust and statistically significant than the findings of a previous study (4). As McEwen recently noted, when facing a stressor, the brain goes through a series of responses that ultimately result in either adaptation (i.e., allostatic load) or pathophysiology (i.e., allostatic overload), or maladaptation (23). Allostatic load could be understood as resilience with the implication that a deficiency of resilience might play a key role in the on-set of mental disorders. Our results go beyond those of McEwen in that they suggest that patients with schizophrenia and bipolar disorder differ from each other and from healthy controls in their level of resilience.

Our study also showed that patients with either schizophrenia or bipolar disorder demonstrated lower levels of cognitive function than healthy controls, including measures of verbal comprehension, executive functioning and working memory. Furthermore, patients with schizophrenia showed significantly lower level of cognitive function compared to patients with bipolar disorder, further demonstrating important differences in severity between these two disorders, an array of differences that suggestively paralleled differences on measures of resilience.

We found significant correlation between resilience and cognitive measures in the whole sample and healthy controls group, while there were no significant correlations in both diagnostic groups. This finding illustrated that in the patients with schizophrenia and bipolar disorder, the severe impairments in resilience and cognitive function might account for not finding significant association of resilience and neurocognition in diagnostic groups.

However, when we further evaluated this suggestive parallelism by conducting a set of linear regressions we did not find evidence that the lack of resilience in clinical groups could be attributed to the observed differences in cognitive ability. These regressions first, showed, as expected a highly significantly negative association between diagnoses of either schizophrenia or bipolar disorder and resilience, net of other sociodemogaphic factors. However, since the mediating effect of neurocognitive functions on the relationship between diagnostic groups and resilience was not significant there were no grounds for concluding that deficits in resilience could be explained by deficits in neurocognition of verbal comprehension, executive functioning and working memory. Several studies have pointed out that resilient individuals tend to possess a positive explanatory style, and strong reappraisal and acceptance when facing adversity, which were identified as features of cognitive functioning, but these studies did not focus on patients with either schizophrenia or bipolar disorder (24–26). Cognitive impairments may also impair process of adaptive emotional regulation (27, 28), the process of regulating emotional responsiveness to stressors, including when and how these emotions are experienced (29), all of which are central to resilience (30, 31). The previous findings suggested a significant association between cognitive function and resilience. However, the analysis of measures of cognitive functioning considered here did not support this perspective in patients with schizophrenia or bipolar disorder. These data may suggest that both resilience and cognitive function are severely impaired in schizophrenia and bipolar disorder patients and that this explained why the mediating effect of neurocognition on the relationship between diagnostic groups and resilience was not significant. It is possible that other measures of cognition, and especially executive function, might have demonstrated a more robust mediating role as Shields et al. and Traub et al. singled out strong executive function as predicting high levels of resilience (32, 33). Measures such as the Wisconsin Card Sorting test (34) or the Trails B test (35), or measures of social cognition (i.e., The Awareness of Social Inferences Test, Penn Emotion Recognition Test) (36) might better identify cognitive functions that mediate resilience. Moreover, patients with schizophrenia and bipolar disorder may have demonstrated low levels of resilience because of their limited functional abilities, as reflected in negative symptoms or lack of motivation which might influence the process of adaptively achieving their goals but might not show up as related to cognition. It is also possible that experiential factors such as traumatic experience or educational deficiencies could better account for deficiencies in resilience among these patients. Further research is thus needed to identify factors that account for that lack of resilience in patients with severe mental disorders.

Several methodological limitations require comment. As noted above the main limitation of this study is that we examined a limited set of measures of cognitive functioning. Second, we didn't address whether patients were clinically stable or in an acute episode, which could have confounded our assessments as acutely disorganized patients may not be able to accurately report on their resilient behaviors. Third, in this study, psychiatric symptoms were assessed only in schizophrenia patients, while depression and mania symptoms were assessed only in bipolar disorder patients. Thus, the influence of the severity of clinical symptoms on the relationship between resilience and cognitive function cannot be ruled out in our analysis. Fourth, while gender, levels of education, marital status and employment were significantly different across groups, we adjusted for these factors which did not affect the significance of differences between groups. Fifth, subjects under 18 years old were recruited, and adolescent might show the different psychopathology, however, patients were matched with healthy controls in the age. Finally, the CD-RISC measure is based entirely on self-report data, whose validity has not been evaluated in Chinese patients with psychotic disorders.

In summary, we found that measures of both resilience and cognitive performance were significantly lower in people diagnosed with schizophrenia than in patients diagnosed with bipolar disorder and both were lower than healthy controls. While suggestive, we found no evidence that compared to healthy individuals, in patients with schizophrenia and bipolar disorder, deficits in resilience were mediated by the measures of cognitive function that we evaluated. These data may suggest that compared to healthy individuals, the impairments in resilience and cognitive function shared different psychopathological pathways. Further research is needed to identify other factors that account for deficiencies in resilience in patients with severe mental disorders.

## Author contributions

MD, WP, and ZL designed the study and wrote the protocol. All authors participated in the data collecting. MD, YP, and RR undertook the statistical analysis. Author MD wrote the first draft of the manuscript. WP, ZL, ZH, and RR modified the manuscript. All authors contributed to and have approved the final manuscript.

### Conflict of interest statement

The authors declare that the research was conducted in the absence of any commercial or financial relationships that could be construed as a potential conflict of interest.
